# Mechanisms of hesperetin in treating metabolic dysfunction-associated steatosis liver disease *via* network pharmacology and *in vitro* experiments

**DOI:** 10.1515/med-2025-1215

**Published:** 2025-06-09

**Authors:** Yi Wu, Ying Lin, Shan Xu, Dan Su, Hao Yang, Lidan Tang

**Affiliations:** Department of Pharmacy, The Second People’s Hospital of Changzhou, The Third Affiliated Hospital of Nanjing Medical University, Changzhou, Jiangsu, 213164, China; Department of Pharmacy, The Second People’s Hospital of Changzhou, The Third Affiliated Hospital of Nanjing Medical University, No. 68. Gehu Middle Road, Changzhou, Jiangsu, 213164, China

**Keywords:** hesperetin, MASLD, IL-6-STAT3-SOCS3 signaling pathway, lipid metabolism, network pharmacology

## Abstract

**Purpose:**

Metabolic dysfunction-associated steatosic liver disease (MASLD) poses a global health challenge with limited therapeutic options. Hesperetin, a flavonoid extracted from citrus, exhibits multiple pharmacological properties, but its mechanisms in MASLD-associated lipid metabolism remain unclear. This study aimed to explore the mechanism of hesperetin for treating MASLD.

**Methods:**

Network pharmacology identified the therapeutic targets for MASLD. Key targets were selected based on network topology analysis. Subsequently, Kyoto Encyclopedia of genes and genomes (KEGG) pathways and gene ontology enrichment were conducted. Molecular docking was performed to evaluate the binding affinity between hesperetin and the identified key targets. *In vitro* validation included lipid accumulation assays and verification of core target modulation *via* western blotting.

**Results:**

Hesperetin significantly attenuated lipid accumulation in free fatty acid -induced HepG2 cells. Forty core targets of hesperetin for MASLD mitigation were identified. Notably, MYC, IL-6, IL1B, and PTGS2 had high network association values. KEGG analysis revealed predominant involvement in cancer-related pathways, non-alcoholic fatty liver disease, and JAK/STAT signaling. Biological processes included inflammatory response regulation and cytokine activity. Molecular docking confirmed strong hesperetin-IL-6 binding. Experimental data suggested that hesperetin may ameliorate lipid accumulation by modulating the IL-6-mediated STAT3-SOCS3 signaling pathway.

**Conclusion:**

Hesperetin may ameliorate MASLD by targeting the IL-6-STAT3-SOCS3 axis, underscoring its therapeutic potential.

## 1 Introduction

Metabolic dysfunction-associated steatosic liver disease (MASLD), previously known as non-alcoholic fatty liver disease (NAFLD), represents the initial stage for developing a malignant cascade characterized by the pathologic accumulation of lipids in hepatocytes [1]. The worldwide prevalence of MASLD exceeds 25%, with approximately 20% of the patients progress to non-alcoholic steatohepatitis and cirrhosis, which eventually leads to hepatocellular carcinoma (HCC) [2,3]. To date, limited reports have elucidated the mechanisms underlying MASLD pathology. Additionally, current treatment options are limited, relying predominantly on lifestyle modifications to attenuate disease progression. Fatty acid transport, synthesis, and β-oxidation processes in hepatocytes are influenced by the pathological conditions of MASLD, resulting in the incomplete breakdown and metabolism of excess fats, causing large amounts of fats. These phenomena trigger substantial fat accumulation in the hepatocytes, which ultimately leads to liver injury [4–6]. Consequently, strategies aimed at reducing hepatic lipid deposition represent a promising approach for the management of MASLD.

Flavonoids possess multiple medicinal properties, particularly decelerating cellular oxidative stress and inflammatory responses [7–10]. Hesperetin (3′, 5′, 7′-trihydroxy-4-methoxyflavone), a natural flavonoid derivative extracted from citrus, is derived from hesperidin through hydrolysis by human intestinal flora. Research indicates numerous therapeutic effects of hesperetin, including antidiabetic, antiviral, antitumor, and neuroprotective properties [11–14]. Studies have shown that hesperetin dose-dependently decreases the activities of alanine aminotransferase (ALT) and aspartate aminotransferase (AST) [15]. Additionally, hesperetin treatment has been found to lower liver injury markers and improve NAFLD by reducing oxidative stress and inflammation [16]. Furthermore, hesperetin regulates hepatic lipid metabolism by influencing key signaling pathways, such as the PI3K-AKT-Nrf2 pathway [16]. However, the effects of hesperetin on MASLD-associated hepatic lipid accumulation and the underlying regulatory mechanisms have not been elucidated completely. Network pharmacology, a systems biology approach, integrates systems biology, bioinformatics, and topological analysis to comprehensively understand the complex mechanisms of small molecules and target proteins in human diseases from a holistic viewpoint [17–19].

**Figure 1:**
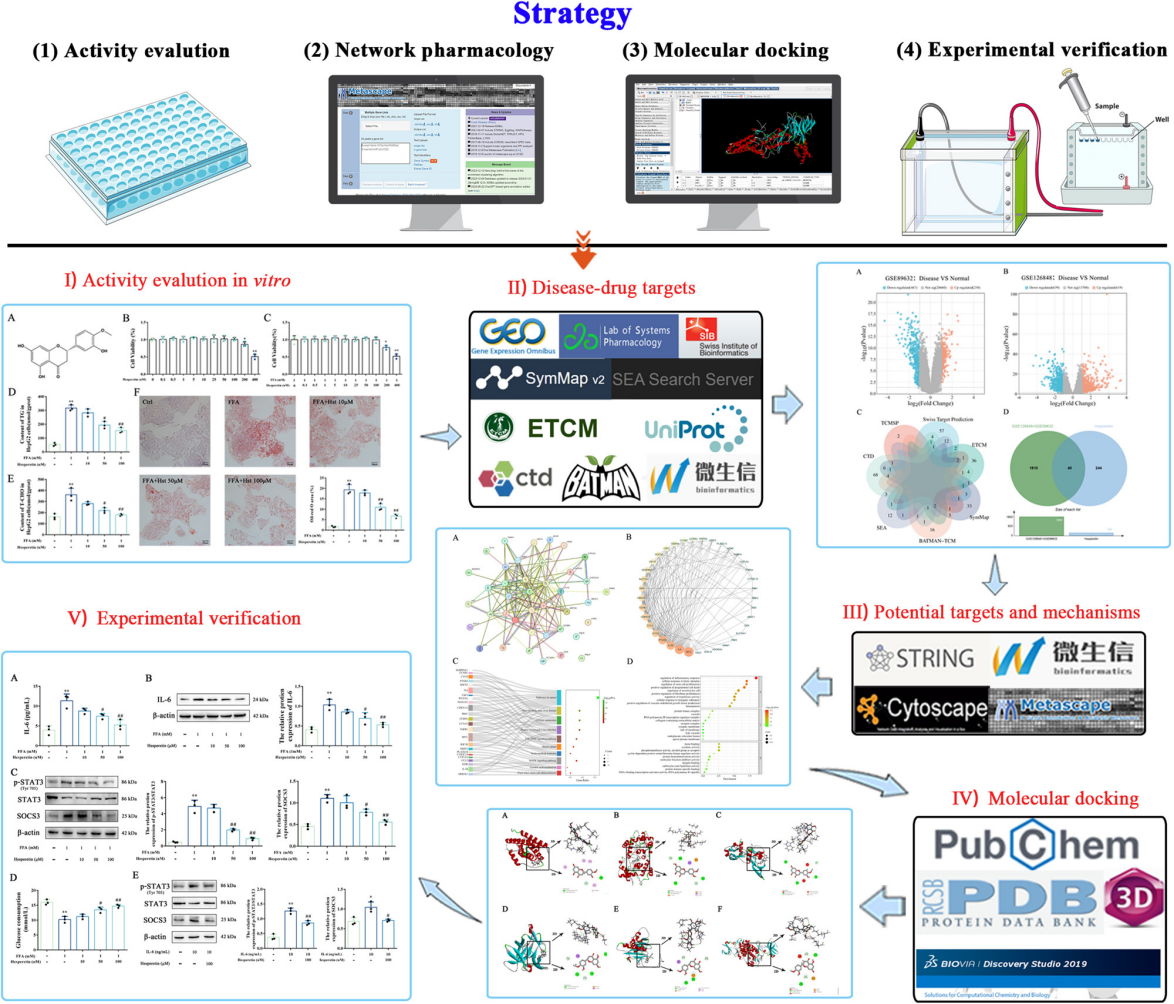
Whole framework of the potential mechanisms of hesperetin on MASLD.

In the present study, we investigated the efficacy of hesperetin in ameliorating abnormal lipid accumulation in MASLD via network pharmacology. Subsequently, we conducted a systematic analysis using several publicly accessible databases, enrichment tools, and bioinformatics resources, combined with comprehensive strategies such as molecular docking and *in vitro* experiments (Figure 1). Overall, this study provided new scientific support for the therapeutic potential and new drug development of hesperetin in MASLD prevention and treatment.

## 2 Materials and methods

### 2.1 Materials

Hesperetin (H107699) was purchased from Aladdin (Shanghai, China). NP-40 (P0013F) and 3-(4,5-dimethiazol-2-yl)-2,5-diphenyl tetrazolium bromide (MTT) (ST1537) were purchased from Beyotime (Nanjing, China). The primary antibodies against interleukin-6 (IL-6) (DF6087), p-JAK2 (AF3024), JAK2 (AF6022), p-STAT3 (AF3293), and STAT3 (AF6294) were purchased from Affinity Biosciences (OH, USA). The primary antibody against β-actin (PAC006) was purchased from Proteinbio (Nanjing, China). Phosphatase inhibitor cocktail (HY-K0021) was purchased from MCE (New Jersey, USA). Free fatty acid (FFA) was purchased from Kunchuang (Xi’an, China). Recombinant human IL-6 (P5138) was purchased from Beyotime (Nanjing, China).

### 2.2 Network pharmacology analysis

#### 2.2.1 Data preparation

First putative hesperetin targets were screened from seven databases, namely the Encyclopedia of Traditional Chinese Medicine (ETCM, http://www.tcmip.cn/ETCM/index.php/Home/), Bioinformatics Annotation Database for Molecular Mechanism of Traditional Chinese Medicine (BATMAN-TCM, http://bionet.ncpsb.org.cn/), Swiss Target Prediction Database (http://www.swisstargetprediction.ch), Traditional Chinese Medicine Systems Pharmacology (TCMSP, https://www.tcmsp-e.com/), Similarity Ensemble Approach Database (SEA, http://sea.bkslab.org), SymMap (http://www.symmap.org/), and Comparative toxicogenomics database (CTD, http://www.ctdbase.org). After that, using the Uniprot database (https://www.uniprot.org/) to obtain the normalization target gene for hesperetin by selecting “*Homo sapiens*.”

Then, the GSE89632 and GSE126848 datasets were retrieved from the Gene Expression Omnibus database (GEO, https://www.ncbi.nlm.nih.gov/). To identify MASLD-related genes, the differently expressed genes among patients with MASLD and healthy individuals were identified using the interactive web tool GEO2R. This step helped us to screen for the genes with substantial differential expression, based on adj.*P*.Val < 0.05 & |log FC| > 1. Additionally, Venn diagrams are drawn to represent the plotted disease–drug sharing targets.

#### 2.2.2 Protein–protein interaction (PPI) network

Gene symbols of hesperetin treatment targets for MSALD were imported into the STRING online database (version 11.5, https://www.string-db.org/). “*Homo sapiens*” was selected as the organism species, to obtain protein interactions among the targets. Subsequently, the PPI networks in tab-separated value format files were imported into Cytoscape 3.7.1. The network topology was analyzed using the network analyzer plug-in; topological features were calculated for each node.

#### 2.2.3 Kyoto Encyclopedia of genes and genomes (KEGG) pathway and gene ontology (GO) enrichment analyses

The intersecting targets underwent KEGG pathway and GO enrichment analyses using the Metascape platform (https://metascape.org/gp/index.html). The threshold value was set at *P*  <  0.05.

#### 2.2.4 Molecular docking

Molecular docking was performed using the LibDock module in Discovery Studio 2019 to evaluate the binding affinity between hesperetin and target proteins [20]. Prior to docking, the 2D structure of hesperetin was acquired in Spatial Data File format from the NCBI PubChem (https://pubchem.ncbi.nlm.nih.gov/), and converted to 3D *via* the “Prepare Ligand” tool with default settings (adding hydrogen atoms, optimizing geometry using the CHARMm force field). Protein crystal structures of the potential targets (*CEBPA*, *IL1B*, *IL-6*, *TGFB1*, *SOCS3*, *CYC3*) were obtained from the Protein Data Bank (PDB) database (https://www.rcsb.org/). PDB IDs of CEBPA, *IL1B*, *IL-6*, *TGFB1*, *SOCS3*, and *CYC3* were 6DC0, 1I1B, 4O9H, 4KV5, 2JZ3, and 3NWV, respectively. The protein structures were prepared by removing water molecules, co-crystallized ligands, and adding polar hydrogens, and optimizing hydrogen-bonding networks *via* the Prepare Protein tool. For the LibDock module, the docking site was defined as the active pocket of each target protein (radius: 10 Å around the co-crystallized ligand). Key parameters included Docking Preferences set to “High Quality,” Hotspot Spacing at 0.5 Å, Ligand Conformations set to 100 poses per run, and Minimization enabled for pose refinement. Binding interactions were analyzed using “Ligand–Protein Interaction” tool, categorizing hydrogen bonds, π–π stacking, and hydrophobic interactions. The highest-scoring pose for each target was chosen based on LibDockScore and structural, ensuring consistency with known functional domains.

### 2.3 Cell culture and treatment

HepG2 was kindly provided by Dr Wang Zeng from Nanjing Medical University. The cells were cultured in Dulbecco’s modified Eagle’s medium, supplemented with 10% fetal bovine serum and 1% penicillin–streptomycin in a humidified incubator at 37°C with 5% CO_2_. The cells were exposed to 1 mM FFA, comprising oleic acid (OA)/palmitic acid (PA) in a 2:1 ratio, consisting of 1% bovine serum albumin for 24 h to simulate the pathological state of MASLD. Subsequently, low (10 μM), medium (50 μM), and high (100 μM) doses of hesperetin (10, 50, and 100 μM) were added for 24 h.

### 2.4 Cell viability assay

HepG2 cells were incubated with hesperetin (0.1, 0.5, 1, 5, 10, 20, 25, 50, 100, 200, and 400 μM) for 24 h. Subsequently, cell viability was monitored using the MTT assay. The percentage of cell viability was computed relative to the absorbance intensity of the control cells.

### 2.5 Western blot

HepG2 cells were lysed with NP-40 lysis buffer (Beyotime, China) and total protein was isolated. Protein concentration was determined using bicinchoninic acid protein assay reagent (Beyotime, China). Total proteins (40 μg) were resolved on 8 or 10% sodium dodecyl-sulfate polyacrylamide gel electrophoresis gel and transferred to nitrocellulose membranes. The membranes were blocked in Tris-buffered saline with 0.1% Tween^®^ 20 detergent (TBST) consisting of 5% skim milk powder for 1 h at room temperature. Furthermore, they were incubated overnight with special antibodies, including IL-6, p-STAT3, STAT3, SOCS3, and β-actin (1:1,000) at 4°C. After washing with TBST three times, the membranes were incubated with secondary antibodies (1:15,000) for 1 h at room temperature.

### 2.6 Cellular triglyceride (TG) and total cholesterol (T-CHO) measurement

TG and T-CHO values in the cell samples were detected by commercial detection kits (Jiancheng Bioengineering Institute, Nanjing, China). After collecting the cells, appropriate amount of phosphate-buffered saline (PBS) was added. The cells were broken by ultrasonication under an ice-water bath. Subsequently, T-CHO and TG levels were detected directly. The absorbance was measured at 500 nm.

### 2.7 Detection of IL-6

Thelevel of inflammatory cytokines IL-6 in cell culture medium was determined using commercial human immunoassay enzyme-linked immunosorbent assay kits (Neobioscience, Shenzhen, China), according to the manufacturer’s instructions.

### 2.8 Quantitative real-time PCR (qRT-PCR)

Total RNA was extracted from FFA-induced HepG2 cells treated with hesperetin (100 μM) using TRIzol reagent (Vazyme Biotech Co. Ltd, Nanjing, China) and quantified. cDNA was synthesized by HiScript II Q RT SuperMix for qPCR (Vazyme Biotech Co. Ltd, Nanjing, China), and qPCR was carried out to amplify the target genes by AceQ Universal SYBR qPCR Master Mix using specific primers (Thermo Fisher, Massachusetts, USA). All gene-specific mRNA expression values were compared to β-actin mRNA levels as a standard (Tongyong Biotech Co. Ltd, Anhui, China). The primer sequences for each gene are listed in Table S1.

### 2.9 Oil-red staining

Oil Red O stain was applied to assess lipid droplet formation in the HepG2 cells [21]. Briefly, HepG2 cells were washed with PBS and fixed with 4% paraformaldehyde for 30 min. The cells were incubated with Oil Red O working solution for 30 min, briefly rinsed in 60% isopropanol, and counterstained with hematoxylin for 1 min. The images were acquired using a microscope (Olympus, Tokyo, Japan).

### 2.10 Glucose consumption assay

HepG2 cells were inoculated in 96-well plates. Glucose uptake was assayed as described previously [20].

### 2.11 Statistical analysis

Data from the three independent experiments are presented as mean ± SD. Statistical analysis was conducted using GraphPad Prism software 6.0. *P*-values ≤0.05 indicated statistical significance.

## 3 Results

### 3.1 Hesperetin alleviated lipid accumulation in FFA-induced HepG2 cells

**Figure 2:**
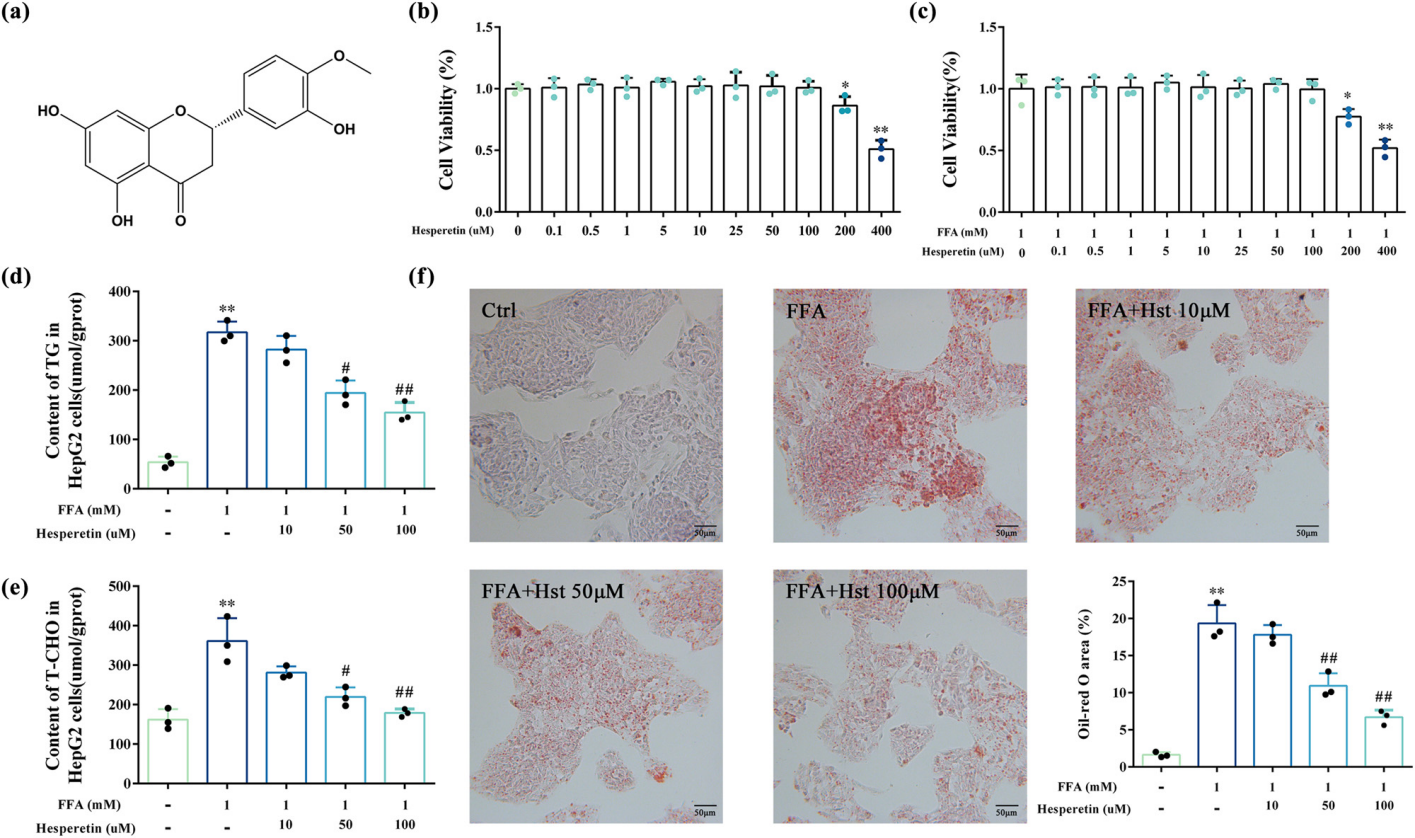
Effects of hesperetin on lipid accumulation in FFA-induced HepG2 cells. (a) 2D structural formula of hesperetin. (b) Effects of different concentrations of hesperetin on cell viability in HepG2 cells. (c) Effects of different concentrations of hesperetin on cell viability in FFA-induced HepG2 cells. (d) Levels of TG in HepG2 cells. (e) Levels of T-CHO in HepG2 cells. (f) Oil Red O staining. Ctrl: HepG2 cells cultured in DMEM; FFA: HepG2 cells treated with FFA; FFA + Hst 10 μM: HepG2 cells treated with FFA + hesperetin 10 μM; FFA + Hst 50 μM: HepG2 cells treated with FFA + hesperetin 50 μM; FFA + Hst 100 μM: HepG2 cells treated with FFA + hesperetin 100 μM. The data represented as mean ± SD, *n* = 3. **P < 0.01, compared to Ctrl; ^#^ *P* < 0.05 or ^##^ *P* < 0.01, compared to FFA.

The anti-MASLD effects of hesperetin were assessed using the FFA-induced HepG2 cell model. The structural formula of hesperetin is shown in Figure 2a. First, the effects of different hesperetin concentrations on the activity of HepG2 cells were evaluated by an MTT assay. At concentrations <100 μM, hesperetin exhibited no significant effects on cell viability (Figure 2b). However, the cell viability was significantly inhibited as the concentration reached 200 or 400 μM. Additionally, we examined the effects of hesperetin on FFA-induced HepG2 cell viability. The cytotoxicity results suggested that hesperetin exerted no significant effect on FFA-induced HepG2 cell viability at concentrations <100 μM (Figure 2c). Thus, hesperetin concentrations of 10, 50, and 100 μM were selected for subsequent experiments. Hesperetin treatment significantly reduced the TG and T-CHO levels in FFA-induced HepG2 cells in a concentration-dependent manner (Figure 2d and e). Oil Red O staining results revealed a dose-dependent decrease in intracellular lipid accumulation with hesperetin treatment, consistent with the TG and T-CHO level findings (Figure 2f). Collectively, the above results supported the potential of hesperetin to ameliorate hepatocyte lipid accumulation in MASLD. Nonetheless, further research is warranted to investigate the precise molecular mechanisms underlying this process.

### 3.2 Exploring hesperetin targets for the MASLD treatment by network pharmacology analysis

**Figure 3:**
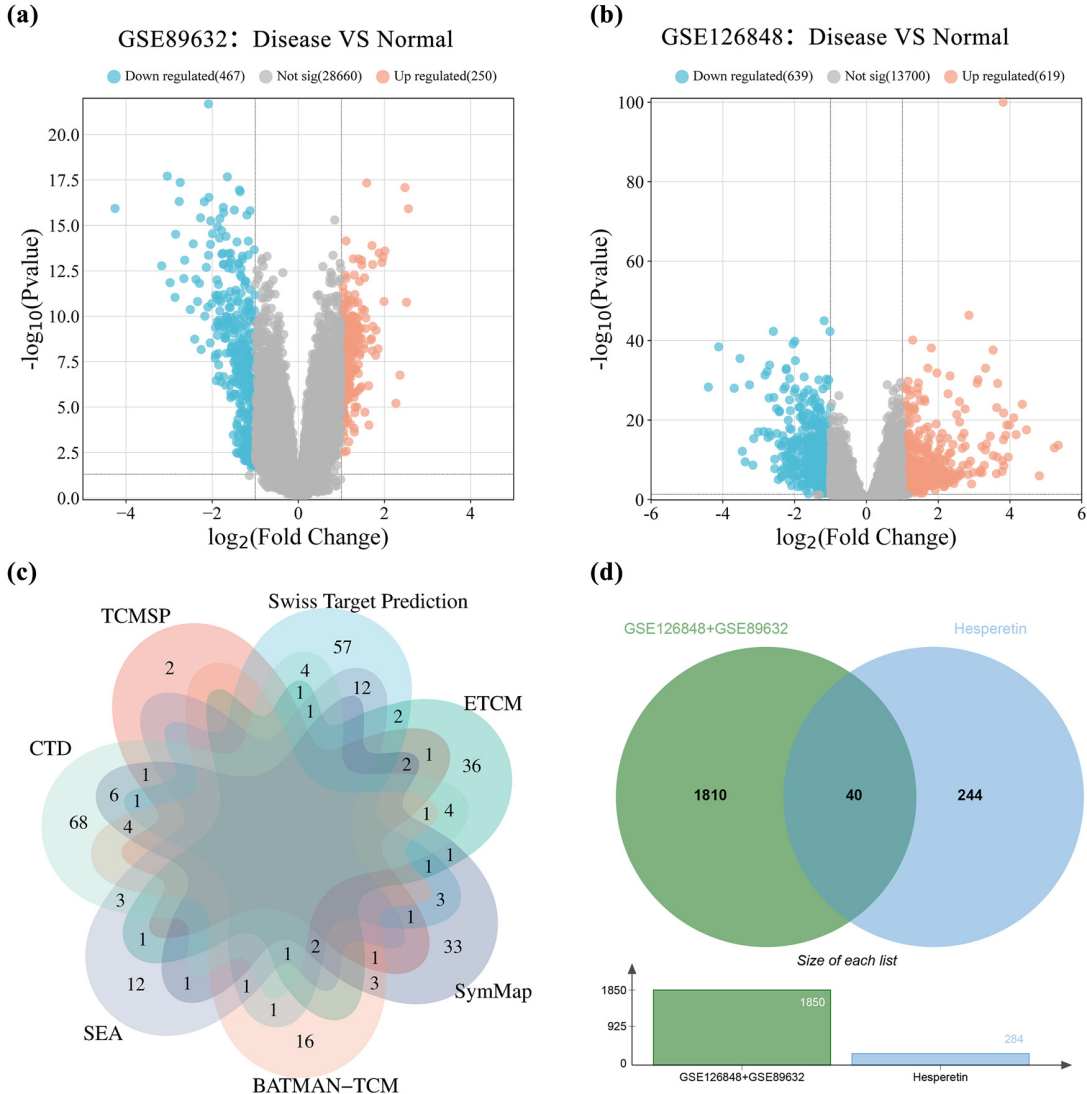
Volcano plot of differentially expressed genes and Venn diagrams of disease–drug intersection targets. (a) Differentially expressed genes in MASLD from GSE89632. (b) Differentially expressed genes in MASLD from GSE126848. (c) Venn diagram of hesperetin targets from different databases. (d) Venn diagram of the intersection of potential target genes of hesperetin and differentially expressed genes in the liver of patients with MASLD.

Figure 1 depicts the workflow for integrating network pharmacology, molecular docking, and experimental validation. Utilizing the GEO database, we obtained 1,850 genes associated with MASLD from the GSE89632 and GSE126848 datasets, following |log FC| > 1 and adj.*P*.Val < 0.05 screening criteria (Figure 3a and b). Subsequently, we retrieved 88, 54, 30, 8, 34, 95, and 60 targets of hesperetin targets from the Swiss target prediction, ETCM, BATMAN-TCM, TCMSP, SEA, CTD, and SymMap databases, respectively. Targets from these seven databases were merged and de-duplicated, resulting in 284 drug targets (Figure 3c). Ultimately, through further integration, we identified 40 overlapping targets corresponding to “Hesperetin-MASLD” (Figure 3d).

### 3.3 PPI analysis for targets of hesperetin against MASLD

**Figure 4:**
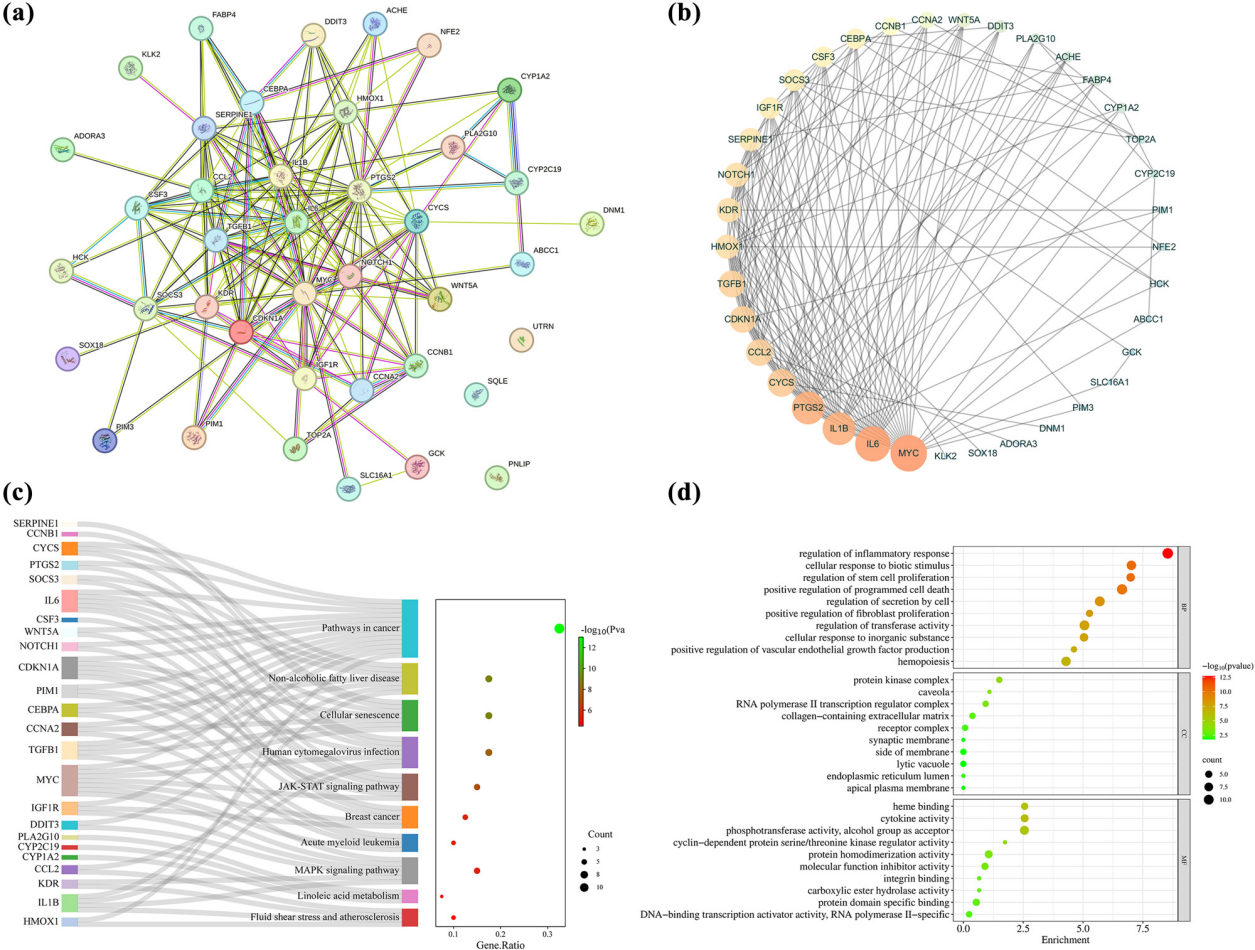
PPI network and enriched GO and KEGG analyses of potential target genes of hesperetin on MASLD. (a) The PPI network analyzed by STRING database. (b) The PPI network analyzed by Cytoscape 3.7.2. (c) KEGG pathway analysis by Sankey diagram. (d) GO functional analysis. The bubble chart shows the GO enrichment terms for BP, CC, and MF.

We constructed a PPI network by importing 40 intersecting targets into the STRING database to explore the mechanism underlying the therapeutic effects of hesperetin on MASLD (Figure 4a). Subsequently, the network was visualized using the Cytoscape 3.7.2 software and subjected to topological analysis (Figure 4b). The center network was based on three topological parameters as follows: betweenness centrality, degree value, and closeness centrality. Table 1 summarizes the topological parameter analysis structure. The leading ten targets were as follows: *MYC* (degree = 24), *IL-6* (degree = 23), *IL1B* (degree = 21), *PTGS2* (degree = 21), *CYCS* (degree = 17), *CCL2* (degree = 16), *TGFB1* (degree = 16), *CDKN1A* (degree = 16), *HMOX1* (degree = 14), and KDR (degree = 14).

**Table 1:** Degree value, betweenness centrality, and closeness centrality of key targets in the PPI network

Gene name	Degree value	Betweenness centrality	Closeness centrality
MYC	24	0.227508	0.75
IL-6	23	0.091871	0.734694
IL1B	21	0.082048	0.705882
PTGS2	21	0.090734	0.705882
CYCS	17	0.084592	0.631579
CCL2	16	0.06862	0.631579
TGFB1	16	0.012414	0.642857
CDKN1A	16	0.026659	0.631579
HMOX1	14	0.034351	0.610169
KDR	14	0.072083	0.610169
NOTCH1	14	0.010395	0.62069
SERPINE1	13	0.061264	0.590164
SOCS3	12	0.033615	0.571429
IGF1R	12	0.051962	0.580645
CSF3	11	0.012691	0.571429
CEBPA	11	0.014722	0.545455
CCNB1	9	0.006468	0.537
CCNA2	8	0.007113	0.529
WNT5A	7	0	0.522
DDIT3	6	0.000894	0.48
ACHE	5	0	0.474
FABP4	5	0.000353	0.474
CYP1A2	5	0.005023	0.474
PLA2G10	5	0.003396	0.468
CYP2C19	4	0.004777	0.444
TOP2A	4	0.000787	0.456
PIM1	3	0.000265	0.45
NFE2	3	0.000198	0.468
HCK	3	0	0.439
ABCC1	2	0.00335	0.456
GCK	2	0.000794	0.379
SLC16A1	2	0.005343	0.444
PIM3	2	0	0.444
ADORA3	1	0	0.391
DNM1	1	0	0.391
SOX18	1	0	0.383
KLK2	1	0	0.375

### 3.4 GO and KEGG pathway enrichment analyses

We performed KEGG pathway analysis on 40 intersecting targets to explore the various mechanisms by which hesperetin action contributes to MASLD treatment. The KEGG analysis examined the pathways central to MASLD pathogenesis. Figure 4c depicts the leading ten enriched pathways with a false discovery rate (FDR) <0.05. Among these pathways, NAFLD, JAK-STAT signaling pathway, and MAPK signaling pathway were highly enriched and met the FDR criteria. Table 2 summarizes the target genes enriched for each pathway.

**Table 2:** KEGG pathways with TOP ten enrichment degree and related gene targets

KEGG pathway	Log10(P)	Target count	Targets
hsa05200: Pathways in cancer	−13.00	13	CCNA2, CDKN1A, CEBPA, HMOX1, IGF1R, IL-6, MYC, NOTCH1, PIM1, PTGS2, TGFB1, WNT5A, CYCS
hsa04932: Non-alcoholic fatty liver disease	−8.89	7	CEBPA, DDIT3, IL1B, IL-6, TGFB1, SOCS3, CYCS
hsa04218: Cellular senescence	−8.89	7	CCNA2, CCNB1, CDKN1A, IL-6, MYC, SERPINE1, TGFB1
hsa05163: Human cytomegalovirus infection	−7.77	7	CDKN1A, IL1B, IL-6, MYC, PTGS2, CCL2, CYCS
hsa04630: JAK-STAT signaling pathway	−7.09	6	CDKN1A, CSF3, IL-6, MYC, PIM1, SOCS3
hsa05224: Breast cancer	−5.84	5	CDKN1A, IGF1R, MYC, NOTCH1, WNT5A
hsa05221: Acute myeloid leukemia	−5.73	4	CCNA2, CEBPA, MYC, PIM1
hsa04010: MAPK signaling pathway	−5.58	6	DDIT3, IGF1R, IL1B, KDR, MYC, TGFB1
hsa00591: Linoleic acid metabolism	−5.07	3	CYP1A2, CYP2C19, PLA2G10
hsa05418: Fluid shear stress and atherosclerosis	−4.47	4	HMOX1, IL1B, KDR, CCL2

Similar to KEGG analysis, we performed a GO enrichment analysis of the 40 intersecting targets; we analyzed biological processes (BP), cellular components (CC), and molecular functions (MF). The leading ten enriched BP, MF, and CC terms are presented as bubble charts (Figure 4d). The GO terms with high enrichment in BP consisted of the regulation of inflammatory response, cellular response to biotic stimulus, protein kinase complex, and cytokine activity.

### 3.5 Analyzing of molecular docking results

**Figure 5:**
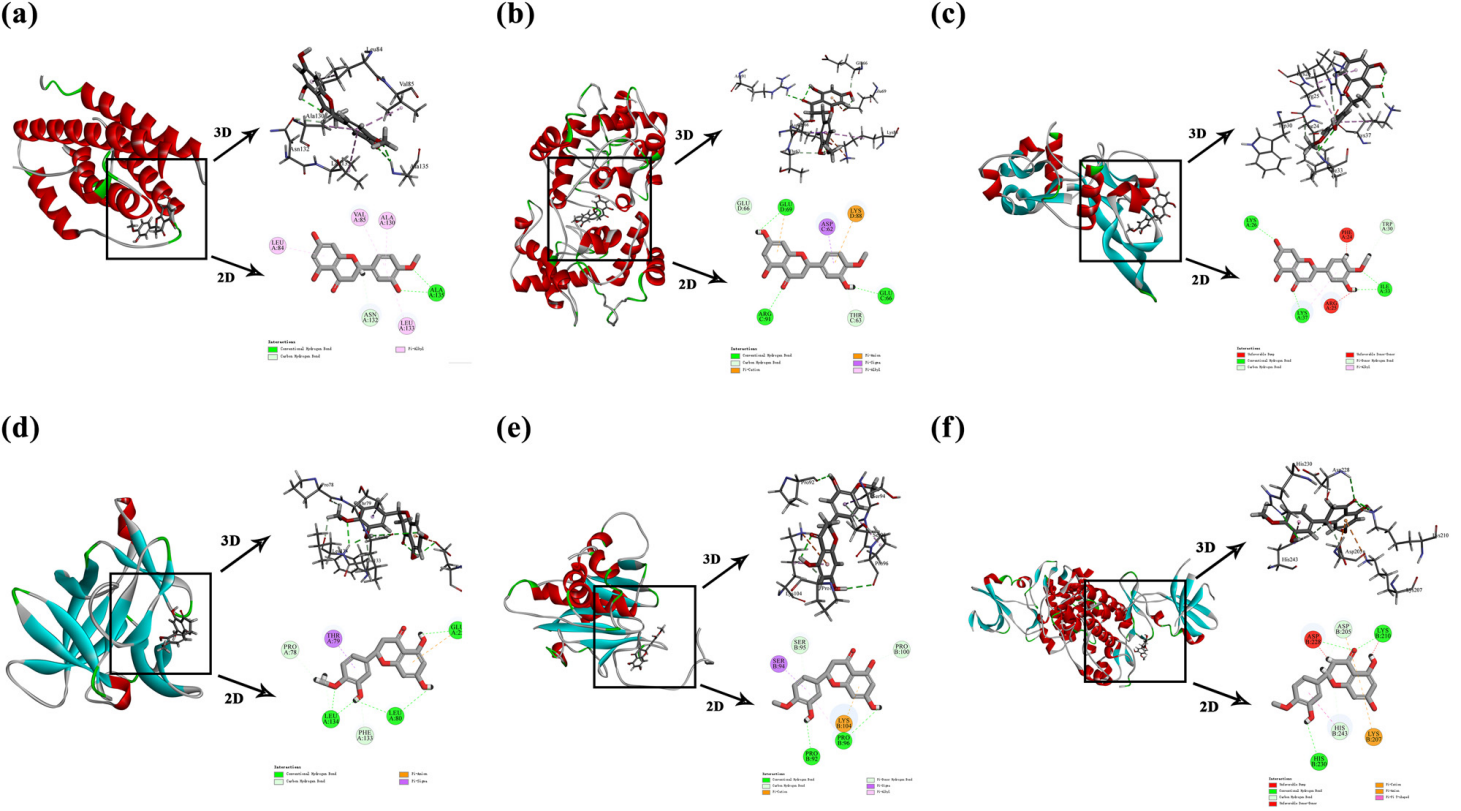
Pattern diagram of molecular docking: (a) Hesperetin-IL-6, (b) hesperetin-CYCS, (c) hesperetin-TGFB1, (d) hesperetin-IL1B, (e) hesperetin-SOCS3, and (f) hesperetin-CEPBA.

Based on the PPI network and KEGG pathway analyses, we selected relevant targets, namely *IL-6*, *CYCS*, *TGFB1*, *IL1B*, *SOCS3*, and *CEPBA*, enriched in the NAFLD pathway for molecular docking. Though DNA damage-inducible transcript 3 (DDIT3) was annotated in the NAFLD pathway, it was excluded from molecular docking experiment because of its low network connectivity (degree = 6) and the lack of a resolved crystal structure, both of which prevented reliable docking analysis. For identifying the interactions, the “LibDock” module in Discovery Studio was used and clusters with the highest LibDockScore were selected (Table 3). Figure 5 presents the corresponding structures. Among these, IL-6 exhibited the highest LibDockScore (113.311), indicating strong binding affinity with hesperetin. This interaction was stabilized by conventional hydrogen bonds, carbon–hydrogen bonds, and pi-alkyl interactions, highlighting structural complementarity. The prioritization of IL-6 aligns with network pharmacology results (high degree value in PPI network, enrichment in NAFLD pathway, and JAK/STAT signaling pathway). As a central proinflammatory cytokine, IL-6 drives JAK/STAT3 signaling, which is dysregulated in MASLD to promote hepatic lipid accumulation and insulin resistance (IR) [22,23]. Other targets such as SOCS3 (LibDockScore = 112.091) showed favorable binding but were functionally linked to IL-6-mediated pathways, reinforcing IL-6 as the pivotal molecular hub. These results provide structural basis for hesperetin’s engagement with the IL-6-STAT3-SOCS3 axis, bridging computational predictions with biological mechanisms. Thus, IL-6 might be a possible key target of hesperetin intervention in MASLD.

**Table 3:** Binding energy and interactions of hesperetin with target proteins

Targets	LibDockScore	Hydrogen bonds and other interacting residues
IL-6	113.311	Ala130, Ala135, Asn132, Leu84, Leu133, Val85
IL1B	77.722	Glu25, Leu80, Leu134, Phe133, Pro78, Thr79
CYCS	101.249	Asp62, Arg91, Glu66, Glu66, Glu69, Lys88, Thr63
TGFB1	96.276	Arg25, Ile33, Lys26, Lys37, Phe24, Trp30
SOCS3	112.091	Lys104, Pro92, Pro96, Pro100, Ser94, Ser95
CEBPA	83.923	Asp205, Asp228, His230, His243, Lys207, Lys210

### 3.6 Hesperetin mitigated hepatocyte lipid accumulation and IR by inhibiting IL-6-STAT3-SOCS3 pathway in FFA-induced HepG2 cells

**Figure 6:**
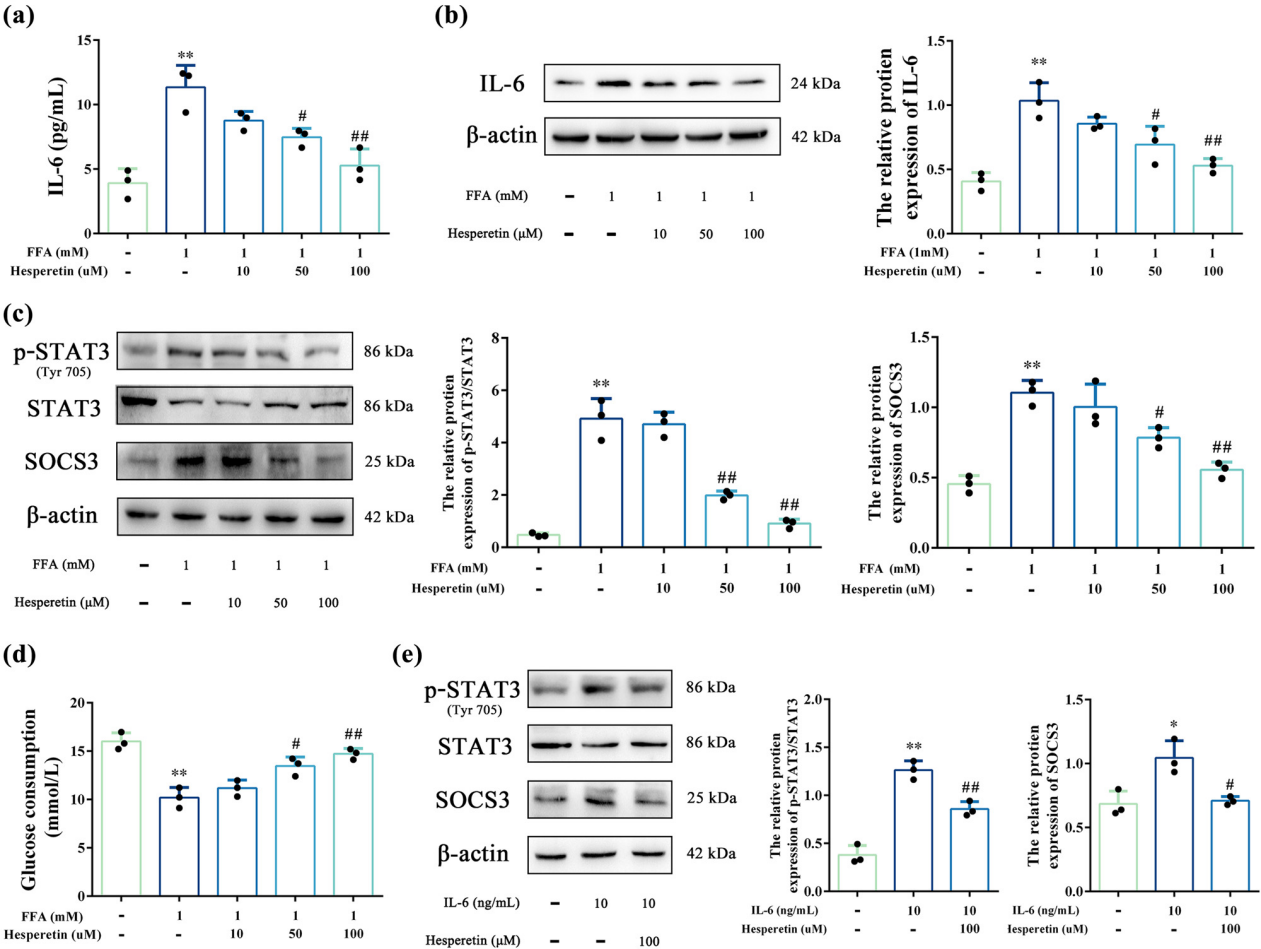
Effects of hesperetin on IL-6-STAT3-SOCS3 pathway in FFA-induced HepG2 cells. (a) IL-6 content in cell culture medium. (b) Relative protein levels of IL-6 in HepG2 cells. (c) Relative protein levels of p-STAT3/STAT3 and SOCS3 in HepG2 cells. (d) Glucose absorption in different groups. (e) Levels of IL-6 in HepG2 cells. (e) Relative protein levels of p-STAT3/STAT3 and SOCS3 in IL-6-induced HepG2 cells. The data represented as mean ± SD, *n* = 3. *P < 0.05, **P < 0.01, compared to Ctrl; ^#^ *P* < 0.05, ^##^ *P* < 0.01, compared to FFA.

Network pharmacological analyses indicated that IL-6 ranked second in the PPI network and exhibited the highest binding affinity with hesperetin, positioning it as a pivotal node linking inflammation (KEGG: JAK-STAT signaling) and metabolic dysfunction (NAFLD pathway). This positioned IL-6 as a pivotal hub linking diverse pathological processes in MASLD. Therefore, we examined the effects of hesperetin on IL-6 secretion in FFA-induced HepG2 cells and the results showed that hesperetin attenuated the IL-6 release from FFA-stimulated HepG2 cells in a dose-dependent manner (Figure 6a). Dysregulated IL-6-STAT3 signaling is directly associated with hepatic lipid accumulation and IR, core features of MASLD. SOCS3, a direct STAT3 target and negative regulator of insulin signaling, was also identified in the docking analysis (LibDockScore = 112.09). We thus further examined the changes in this signaling axis, and western blot results showed that hesperetin inhibited IL-6 protein expression (Figure 6b). Furthermore, p-STAT3 and SOCS3 expressions were significantly increased in FFA-induced HepG2 cells than in controls. However, p-STAT3 and SOCS3 expression decreased significantly after hesperetin treatment than after no-treatment (Figure 6c). Additionally, glucose consumption by FFA-induced HepG2 cells was significantly low. Compared with the low-dose group, the high-dose group demonstrated significant improvement in glucose consumption, suggesting that hesperetin reduced IR and promoted glucose uptake (Figure 6d).

To further validate the critical role of hesperetin in the IL-6-mediated STAT3-SOCS3 pathway, the IL-6-stimulated HepG2 cells was carried out. The results of western blot demonstrated that p-STAT3/STAT3 and SOCS3 levels were significantly elevated after IL-6 stimulation compared to the control. In contrast, the levels were significantly decreased after hesperetin treatment compared with IL-6 stimulation (Figure 6e).

### 3.7 Hesperetin regulates mRNA expression of key PPI network targets

To comprehensively assess the pharmacological network of hesperetin, we examined its effects on mRNA levels of genes enriched in the top five-degree values in the PPI network and in the KEGG “non-alcoholic fatty liver” pathway (MYC, IL-6, IL1B, PTGS2, CYCS, TGFB1, CEBPA, DDIT3, and SOCS3) in FFA-induced HepG2 cells using qRT-PCR (Figure S1). Compared to the FFA group, hesperetin (100 μM) significantly downregulated the mRNA expression of MYC, IL-6, IL1B, PTGS2, CYCS, DDIT3, and SOCS3. In contrast, other genes showed no significant changes, suggesting tissue-specific or compensatory regulatory mechanisms. These findings validate the predictions of network pharmacology and demonstrate that hesperetin engages a network of targets spanning inflammation, metabolism, and apoptosis, reinforcing its polypharmacological action in MASLD.

## 4 Discussion

MASLD is one of the most prevalent types of liver disease worldwide; it is characterized by abnormal lipids accumulation in the liver, which can eventually lead to cirrhosis and HCC [24]. No approved drugs are available for the treatment of MASLD. Lifestyle and dietary interventions remain the only recognized treatment options [25]. Hesperetin exerts therapeutic effects on numerous diseases as a natural product, characterized by multi-target and multi-pathway regulation; however, the exact mechanism by which it ameliorates hepatic abnormal lipid accumulation warrants further investigation [26–28]. In this study, we integrated network pharmacology, molecular docking, and *in vitro* experiments to explore the potential mechanism of hesperetin against MASLD.

Hesperetin, a flavonoid extracted from citrus species with high bioavailability, has been extensively evaluated for its wide range of pharmacological activities, such as antioxidant, anti-inflammatory, anti-diabetes, and anti-cancer properties [28,29]. Notably, its therapeutic potential is further supported by favorable pharmacokinetic profiles, as demonstrated in a clinical study involving six healthy volunteers who received 135 mg oral hesperetin. The results demonstrated that hesperetin was rapidly absorbed, with the plasma concentration peaking after 20 min; in addition, the area under the plasma concentration–time curve extrapolated to the time to infinity (AUC_0−∞_), the elimination half-life (*t*_1/2_), and the maximum (*C*_Max_) were 4846.20 ± 1675.99 ng h/mL, 3.05 ± 0.91 h, and 825.78 ± 410.63 ng/mL, respectively [30]. Notably, findings from rodent models demonstrated preferential hepatic accumulation of hesperetin, with hepatic concentrations exceeding those in extrahepatic tissues (e.g., aorta and kidney) [31]. Collectively, these pharmacokinetic traits underscore hesperetin’s suitability for modulating liver-specific pathologies such as MASLD. Previous studies have shown that hesperetin attenuated OA or PA-induced hepatotoxicity and oxidative stress; nonetheless, there are limited data on the *in vitro* hypolipidemic activity of hesperetin [16,32]. Therefore, we investigated the hypolipidemic activity of hesperetin in FFA-induced HepG2 cells. After determining the hesperetin dose, we investigated the effects of hesperetin on FFA-induced lipid accumulation in HepG2 cells. Oil-red O staining results and biochemical indices reflected that hesperetin significantly reduced aberrant intracellular lipid accumulation in the model group. This finding indicated that hesperetin has the potential to improve hepatocyte lipid metabolism in MASLD.

Through network pharmacology analysis, we identified 40 putative hesperetin-MASLD targets. To explore the core targets, we constructed a PPI network expressing protein interactions by the STRING database and Cytoscape software; *MYC*, *IL-6*, *IL1B*, *PTGS2*, *CYCS*, *CCL2*, *CDKN1*, *TGFB1*, *HMOX1*, and *KDR* might be the possible core targets warranting attention. These targets are closely related to oxidative stress, inflammatory response, and lipid metabolism. For instance, *MYC*, with the highest degree value, plays a crucial role in hepatocyte apoptosis, liver fibrosis, and HCC in the MASLD mouse model [33,34]. *IL-6* and *IL1B* are important promoters of the inflammatory response, which can accelerate the progression of MASLD. [35]. *PTGS2*, also termed cyclooxygenase 2, is expressed predominantly in inflammatory cells. It is substantially upregulated in chronic and acute inflammation, and, thus is a key target for several pharmacological inhibitors [20,36].

KEGG pathway and GO enrichment analyses provided further insights into the underlying mechanisms. GO enrichment analysis demonstrated that several BP are associated with the role of hesperetin in ameliorating MASLD. The regulation of inflammatory response, cellular response to biotic stimulus, regulation of stem cell proliferation, positive regulation of programmed cell death, and regulation of secretion were considerably enriched in BP. Additionally, the target genes were predominantly enriched in cytokine activity, phosphotransferase activity, and protein kinase complex, which demonstrated a potential relationship between hesperetin and the 40 MASLD targets. According to KEGG pathway analysis, significantly enriched pathways associated with MASLD encompassed cancer, NAFLD, cellular senescence, human cytomegalovirus infection, JAK-STAT signaling pathway, and MAPK signaling pathway. The JAK-STAT pathway is central to cytokine-mediated inflammation and serves as a key bridge between immune responses and metabolic dysfunction in MASLD. Activation of JAK-STAT by pro-inflammatory cytokines promotes STAT3 phosphorylation, leading to the upregulation of inflammation-related genes like SOCS3, which in turn impairs insulin signaling and results in hepatic lipid accumulation [37]. Meanwhile, the NAFLD pathway, characterized by dysregulated lipid metabolism and oxidative stress, further exacerbates JAK-STAT activation through an inflammatory feedback loop [38]. The MAPK pathway, responsible for cellular stress and proliferation, interacts with the JAK-STAT and NAFLD pathways to modulate MASLD progression. The phosphorylation cascade of ERK1/2 and p38 within the MAPK axis enhances the transcription of pro-inflammatory cytokines, thereby amplifying JAK-STAT activation [39]. Our *in vitro* experiments demonstrated that hesperetin may disrupt this vicious cycle by inhibiting IL-6-mediated STAT3 phosphorylation, thereby attenuating inflammatory signaling in the JAK-STAT axis and lipid dysregulation in the NAFLD pathway. These results suggested that hesperetin may exert its therapeutic effects on MASLD through multi-pathway regulation.

To explore the binding mode and ability of hesperetin to potential MASLD targets, we selected core targets enriched in the NAFLD signaling pathway for molecular docking with hesperetin based on KEGG analysis results. Molecular docking analysis revealed that hesperetin exhibited stable binding interactions with potential MASLD targets, particularly demonstrating a high binding affinity with IL-6. While the PPI network and molecular docking identified multiple potential targets for hesperetin, our decision to focus on the IL-6-STAT3-SOCS3 axis was based on three key factors aligning with the study’s mechanistic goals and clinical significance. First, IL-6 ranked second in the PPI network, followed only by MYC, and exhibited the highest binding affinity with hesperetin among all validated targets. Second, IL-6 as a pivotal node linking two hallmark pathways of MASLD: inflammation (JAK-STAT signaling) and metabolic dysfunction (NAFLD pathway). Lastly, SOCS3, a direct downstream target of STAT3, also demonstrated strong docking affinity with hesperetin, further emphasizing its significance as a crucial effector molecule. To validate this, we conducted *in vitro* experiments and found that hesperetin attenuated the IL-6 release from FFA-stimulated HepG2 cells in a dose-dependent manner. Moreover, western blot analysis showed that hesperetin treatment significantly decreased the expression of p-STAT3 and SOCS3 in FFA-induced HepG2 cells. Notably, hesperetin has demonstrated significant inhibitory effect on IL-6 in different disease models. Zhang et al. demonstrated that hesperetin significantly alleviated dextran sodium sulfate-induced colitis symptoms and inhibited IL-6 expression both *in vivo* and *in vitro* [40]. Moreover, it exerted anti-neuroinflammatory effects by inhibiting microglia activation and downregulating the mRNA transcript levels of inflammatory cytokines, such as tumor necrosis factor-alpha and IL-6 [41]. Consistent with our findings, Dr Sun reported on the lipid-lowering ability of hesperetin and showed that hesperetin inhibited inflammatory responses in OA-induced HepG2 cells and high-fat diet (HFD)-induced mice [16]. The JAK/STAT pathway plays a crucial role in lipid metabolism and inflammation [42,43]. Dysregulation of the JAK/STAT signaling pathway leads to numerous diseases, including liver disease [44]. Cytokine-induced activation of the JAK/STAT pathway in the liver, notably by IL-6, triggers increased phosphorylation of STAT3, leading to IR and abnormal lipid accumulation [45]. Additionally, SOCS3 is closely related to IR development, as its overexpression in the liver leads to IR, while its inhibition enhances insulin sensitivity and ameliorates hepatic steatosis [46,47]. Cytokines upregulate SOCS3 transcription, primarily through STAT3 activation [48]. Taken together, hesperetin attenuated hepatocellular steatosis by inhibiting IL-6 expression and secretion, thereby blocking STAT3 phosphorylation and SOCS3 levels. While this focus on IL-6 enabled rigorous validation of a biologically plausible pathway, we recognize that other targets within the PPI network (such as MYC, IL1B, PTGS2) could also contribute to the effects of hesperetin. The validation of MYC, IL1B, and PTGS2 through qRT-PCR further supports the predictions of network pharmacology. The downregulation of IL1B and PTGS2 aligns with their roles in amplifying inflammatory cascades, reinforcing hesperetin’s pleiotropic anti-inflammatory action. In addition, while this study establishes an association between hesperetin and the modulation of IL-6-STAT3-SOCS3 signaling, further confirmation of causality is necessary. More genetic interventions such as IL-6 knockdown or SOCS3 overexpression in hepatocytes would enhance the mechanistic understanding. Without these additional experiments, the exact role of hesperetin in modulating this pathway remains to be further verified. Additionally, *in vivo* studies are needed to confirm the translational relevance of these findings.

This study also has several limitations that need to be addressed. First, challenges persist in the accuracy and reliability of retrieving data on compounds and disease targets from various databases due to their lack of comprehensiveness and currency. Moreover, it lacked *in vivo* and clinical experimental studies to directly validate the anti-MASLD effects of hesperetin and its associated pathways. Previous studies have demonstrated the hepatoprotective effects of hesperetin in OA-induced HepG2 cells and HFD-induced NAFLD rat models [16]. Furthermore, studies in different disease models have explored the biological effects of hesperetin on IL-6 gene expression and STAT3 phosphorylation signaling pathway [49,50]. However, in-depth *in vivo* and clinical studies are indispensable. Additionally, more research is required to determine the optimal dose of hesperetin for clinical treatment, as well as the potential effects of long-term hesperetin treatment on other organs and tissues.

## 5 Conclusion

In summary, we systematically explored the pharmacological mechanism by which hesperetin alleviated MASLD using a comprehensive strategy of network-based computational pharmacology, along with bioinformatics analysis. We identified 40 targets on MASLD for hesperetin treatment, of which IL-6 was a possible core therapeutic target. Furthermore, the *in vitro* validation experiments suggested that hesperetin may alleviate FFA-induced lipid accumulation partially through modulation of the IL-6-mediated STAT3-SOCS3 signaling pathway, warranting further mechanistic validation. Taken together, we hypothesized that hesperetin may be a potential therapeutic agent for MASLD.

## Abbreviations

ALT: alanine aminotransferase
AST: aspartate aminotransferase
BATMAN-TCM: Bioinformatics Annotation Database for Molecular Mechanism of Traditional Chinese Medicine
BP: biological processes
CC: cellular components
CTD: Comparative Toxicogenomics Database
DDIT3: DNA damage-inducible transcript 3
ETCM: Encyclopedia of Traditional Chinese Medicine
FDR: false discovery rate
FFA: free fatty acid
GEO: Gene Expression Omnibus
GO: gene ontology
HCC: hepatocellular carcinoma
HFD: high-fat diet
IL-6: interleukin-6
IR: insulin resistance
KEGG: Kyoto Encyclopedia of genes and genomes
MASLD: metabolic dysfunction-associated steatosic liver disease
MF: molecular functions
MTT: 3-(4,5-dimethiazol-2-yl)-2,5-diphenyl tetrazolium bromide
NAFLD: non-alcoholic fatty liver disease
OA: oleic acid
PA: palmitic acid
PBS: phosphate-buffered saline
PDB: Protein Data Bank
PPI: protein–protein interaction
SEA: Similarity Ensemble Approach Database
T-CHO: total cholesterol
TBST: Tris-buffered saline with 0.1% Tween^®^ 20 detergent
TCMSP: Traditional Chinese Medicine Systems Pharmacology
TG: triglyceride

## Supplementary Material

Supplementary material
