# Supplementary material for: Mechanisms of hesperetin in treating metabolic dysfunction-associated steatosis liver disease via network pharmacology and in vitro experiments
Source: Open Med (Wars). 2025 Jun 9;20(1):20251215. doi: 10.1515/med-2025-1215 (PMC12163577; doi:10.1515/med-2025-1215)
Supplement: Supplementary material [file med-2025-1215-sm.pdf]

Supplementary material

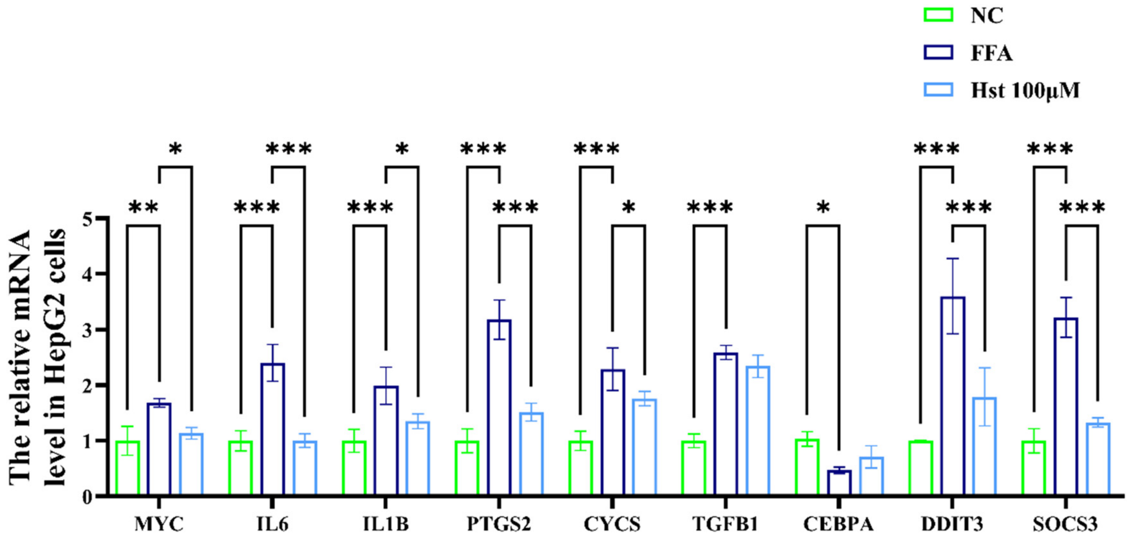

Figure S1: The relative mRNA levels of MYC, IL6, IL1B,PTGS2, CYCS, TGFB1, CEBPA, DDIT3, and SOCS3 in HepG2 cells. The data represented as the mean ± SD, n = 3. $^{*}P < 0.05$, $^{**}P < 0.01$, $^{**}P < 0.001$.

Table S1: Primer sequences for diferent genes of HepG2 cells

Gene	Forward (5′-3′)	Reverse (5′-3′)
MYC	GGCTCCTGGCAAAAGGTCA	CTGCGTAGTTGTGCTGATGT
IL6	ACTCACCTCTTCAGAACGAATTG	CCATCTTTGGAAGGTTCAGGTTG
IL1B	ATGATGGCTTATTACAGTGGCAA	GTCGGAGATTCGTAGCTGGA
PTGS2	CTGGCGCTCAGCCATACAG	CGCACTTATACTGGTCAAATCCC
CYCS	CTTTGGGCGGAAGACAGGTC	TTATTGGCGGCTGTGTAAGAG
TGFB1	GGCCAGATCCTGTCCAAGC	GTGGGTTTCCACCATTAGCAC
CEBPA	GTGGACAAGAACAGCAACGA	GGTCATTGTCACTGGTCAGC
DDIT3	GGAAACAGAGTGGTCATTCCC	CTGCTTGAGCCGTTCATTCTC
SOCS3	CCTGCGCCTCAAGACCTTC	GTCACTGCGCTCCAGTAGAA
β-actin	ATGTGGATCAGCAAGCAGGA	AAGGGTGTAAAACGCAGCTCA
